# Awareness of Molar Incisor Hypomineralisation (MIH) and Hypomineralised Second Primary Molars (HSPMs) among Northern Italian Dentists: A Questionnaire Survey

**DOI:** 10.3390/dj12080271

**Published:** 2024-08-22

**Authors:** Elena Bardellini, Giulio Conti, Federica Veneri, Alessandra Majorana, Francesca Amadori

**Affiliations:** 1 Department of Medical and Surgical Specialties, Radiological Sciences and Public Health, School of Pediatric Dentistry, University of Brescia, Pl. Spedali Civili 1, 25123 Brescia, Italy; alessandra.majorana@unibs.it (A.M.); francesca.amadori@unibs.it (F.A.); 2 Department of Medicine e Surgery, School of Dentistry, University of Insubria, Via Ravasi 2, 21100 Varese, Italy; giulio.conti@uninsubria.it; 3 Department of Surgery, Medicine, Dentistry and Morphological Sciences with Transplant Surgery, Unit of Dentistry & Oral-Maxillo-Facial Surgery, University of Modena and Reggio Emilia, Via del Pozzo, 41124 Modena, Italy

**Keywords:** developmental enamel defects, HSPM, hypomineralisation, MIH, survey

## Abstract

Background: The purpose of this study was to assess the awareness of molar incisor hypomineralisation (MIH) and hypomineralised second primary molars (HSPMs) among dental practitioners in Northern Italy, and to gather data on the occurrence of these conditions in their paediatric patients. Methods: A cross-sectional study was conducted using a structured online questionnaire administered through Google Forms. The survey comprised 10 single-choice questions addressing the occurrence of MIH and HSPM in caries-free patients aged 6–9 years. Results: A total of 315 dentists participated in the survey, yielding a response rate of 31.5%. The majority of respondents reported that 1–25% of their paediatric patients exhibited HSPMs. Among this group, 43.62% of respondents observed MIH in 1–25% of cases. Conclusion: The findings from this survey suggest a consistent perception of MIH and HSPMs among dental practitioners, aligning with known prevalence data and indicating recognition of these conditions within the dental community in Northern Italy.

## 1. Introduction

In recent decades, a substantial body of literature has extensively documented developmental enamel defects (DDEs) impacting both primary and permanent teeth. These defects involve alterations in the quality and/or quantity of dental enamel due to disturbances during amelogenesis [1,2]. Molar incisor hypomineralisation (MIH), officially defined in 2001 and endorsed by the European Academy of Paediatric Dentistry (EAPD), delineates a specific hypomineralisation pattern, primarily affecting at least one first permanent molar and often extending to associated permanent incisors [3,4,5,6,7]. In 2008, Elfrink et al. introduced the term “hypomineralized second primary molars (HSPM)” for DDEs observed on second primary molars [8].

Teeth affected by MIH exhibit demarcated white, yellow, or brown opacities, causing aesthetic concerns, hypersensitivity, inflammation, tooth wear, post-eruptive breakdown, and an elevated risk of caries, significantly impacting the oral health-related quality of life in children [9,10].

In comparison to healthy teeth, teeth with MIH exhibit a histologically prism sheath that is less distinct. This defect is characterised by a scarcity of hydroxyapatite crystals [11]. The hypomineralised enamel possesses diminished mechanical properties, including lower hardness and elasticity, in contrast to sound enamel [11]. Furthermore, enamel affected by these conditions displays an increased presence of proteins such as serum albumin, type I collagen, ameloblastin, a1-antitrypsin, and antitrobin III. These proteins inhibit the growth of hydroxyapatite crystals, leading to a reduction in enamel minerals [11].

The etiology of MIH is multifactorial, encompassing genetic, iatrogenic, perinatal, and pregnancy-related conditions, diseases, and environmental contaminants [12,13]. A review by Butera underscores the complexity of these pathologies, summarising numerous studies exploring environmental or genetic factors, often yielding inconclusive findings [14].

The maturation period of enamel affected by defects in primary and permanent molars spans from the last trimester of pregnancy to the third year of a child’s life [14]. During this period, genetic variations may interact with environmental factors. Prenatal, perinatal, and post-natal factors can influence enamel structure, hindering enzyme action and crystal development [14]. While various factors, such as smoking and alcohol consumption during pregnancy, premature birth, perinatal complications, hypoxia, low birth weight, calcium metabolic disorders, prolonged breastfeeding, childhood diseases, high fever episodes, use of antibiotics/drugs, asthma, and respiratory problems, have been extensively investigated, a definitive cause remains unidentified [12,13,14].

An association between dental caries and enamel hypomineralisation has also been established [15,16]. A systematic review [15] indicated that the probability of finding a child with both MIH and dental caries is 2.1–4.6 times higher than finding a child with MIH without dental caries. Proposed explanations for this association include alterations in the structure and composition of hypomineralised teeth, such as increased porosity, higher carbon and carbonate content, and the presence of posteruptive enamel breakdown. However, the relationship between hypomineralisation and the activity of the dental caries lesion has been poorly investigated [15,16].

Management strategies involve preventive measures, including oral hygiene, diet, re-mineralising agents, desensitising agents, and appropriate fissure sealants. In severe cases, restorative options encompass bioactive, glass ionomer cement-based, hydrophilic, or traditional resin-based materials [17,18]. MIH and HSPMs share similar clinical features, including demarcated opacities and atypical dental caries lesions/restorations [6]. Recent studies highlight the association between HSPMs and MIH [14,15,18]. Garot et al.’s [19] systematic review demonstrated HSPM’s higher probability of presenting MIH, suggesting a potential predictive role of HSPMs. Quintero [20] illustrated a strong association between MIH and HSPMs, emphasising the role of the dental caries lesion in influencing the strength of this association and the severity of the defects.

The reported prevalence of MIH spans a considerable range, fluctuating from 2.9 to 44%, with a specific estimate centring around 13.5%. HSPMs are identified in approximately 3.6% of MIH cases, presenting an overall lower prevalence that falls within the range of 0 to 21.8%. Notably, HSPMs often manifest in milder forms compared to MIH [21,22,23,24]. The co-occurrence of HSPMs and MIH displays geographical variability and is influenced by a spectrum of factors, encompassing environmental, genetic, health-related, and lifestyle considerations [20].

Dental practitioners’ awareness of emerging dental pathologies plays a crucial role in the diagnosis, treatment, and management of these conditions. Given the relatively recent recognition of MIH and HSPMs, there may be varying levels of perception and understanding among dentists [25]. Perception encompasses the awareness of prevalence and possible aetiology, and a recognition of clinical signs and symptoms and appropriate management strategies. A comprehensive understanding of these emerging pathologies is essential for providing optimal care to patients, emphasising the importance of continuous education and awareness initiatives within the dental community. In light of these considerations, this study aims to investigate MIH and HSPM perception by dental practitioners in Northern Italy, with the objective of elucidating practitioners’ awareness regarding these conditions.

## 2. Materials and Methods

### 2.1. Questionnaire

A cross-sectional study was designed as a survey to collect data on molar incisor hypomineralisation (MIH) and hypomineralised second primary molar (HSPM) perception of Northern Italian dentists in their paediatric patients. A structured online questionnaire was administered through Google Forms (Google LLC, Mountain View, CA, USA). No specific approval for this type of study was required from the local ethics committee. The University of Brescia was responsible for data collection and management. The questionnaire was directed at Italian dentists and distributed through the authors’ mailing list, the local national dental association (ANDI Brescia), and social networks. Participation in the questionnaire was voluntary and anonymous, and came without any form of remuneration. The invitation email outlined the research objectives and indicated that the project and its results would be disseminated through scientific publications.

The questionnaire, formulated in the Italian language, comprised 10 single-choice questions (see Supplementary Materials). The queries addressed the percentage of children among the total number of annual patients, the proportion of paediatric patients aged 6 to 9 years, and the percentage of children with opacities on second primary molars and/or first permanent molars among caries-free children aged 6 to 9 years. The survey focused on caries-free children to specifically assess enamel defects and reduce potential bias.

The questionnaire underwent a comprehensive validation process that included both internal and external assessments. Internally, we conducted a pilot test with a small group of five dental professionals from the dental clinic to identify any ambiguities, inconsistencies, or areas requiring clarification. Externally, the pilot test was distributed to ten randomly selected dentists from the target population to further assess the questionnaire’s effectiveness and suitability. This external validation process was instrumental in refining the questionnaire and ensuring its appropriateness for the intended study population.

### 2.2. Sample Size

The sample size was determined a priori, considering a target population of 1000 dentists, a confidence level of 95%, and a margin of error of 5%, requiring a sample size of 100 respondents.

### 2.3. Statistical Analysis

The collected data were exported to an Excel spreadsheet (Microsoft Corp., Redmond, WA, USA) and analysed using STATA17 (Stata Corp., College Station, TX, USA). Categorical data were processed using descriptive statistics. Absolute and relative frequencies of the answer options were calculated for the individual questions and presented in tabular form. Data amenable to comparison (i.e., presence or absence of a characteristic) were assessed using the chi-square test; a *p*-value < 0.05 was considered statistically significant.

## 3. Results

### 3.1. Characteristics of Respondents

**Figure 1:**
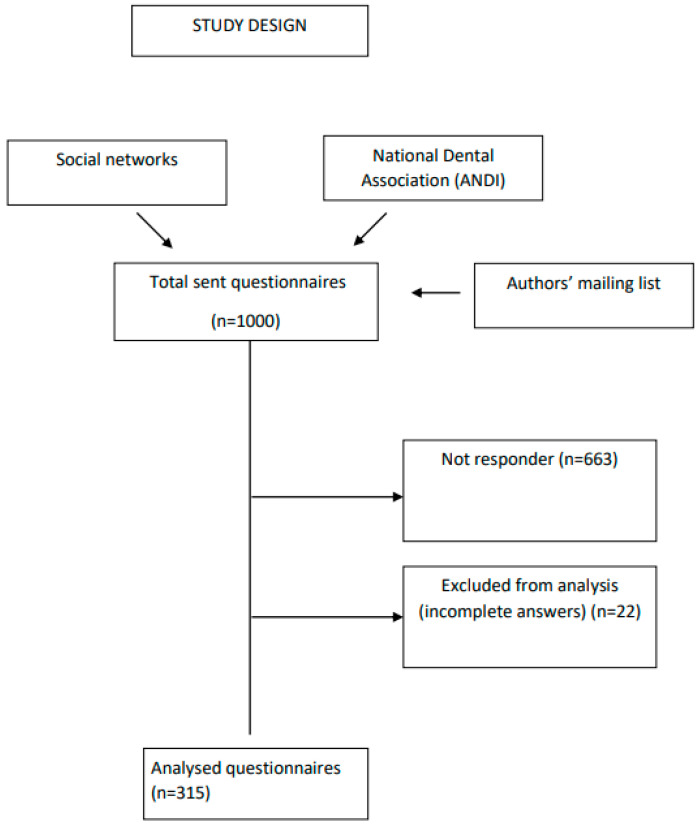
Study design diagram.

Three hundred and fifteen dentists actively participated in the survey, reflecting a response rate of 31.50%, considering the initial pool of 1000 invited professionals (Figure 1). The characteristics of respondents are displayed in Table 1.

Notably, the majority of respondents (83.49%) reported treating fewer than 100 paediatric patients annually. In further detail, 11.43% of participants handled a patient load ranging between 101 and 200 children, while a smaller percentage (5.08%) managed more than 200 paediatric patients.

In terms of the demographic composition, paediatric patients constituted up to 25% of the annual patient pool for 82.22% of the surveyed dentists. Specifically focusing on children aged 6–9 years, approximately 55.24% of respondents indicated that this age group represented less than a quarter of their annual paediatric patients, while 40.63% reported a range of 26–50%. Examining caries prevalence in children aged 6–9 years, 71.75% of dentists reported encountering less than 25% of caries-free cases, and 10.48% identified a prevalence falling between 26 and 50%.

Regarding recall practices, nearly half of the participants (47.94%) scheduled follow-up appointments for paediatric patients every six months, with 23.81% opting for a more frequent interval of every three months. Conversely, a minor fraction (10.16%) of respondents reported seeing paediatric patients primarily for urgent treatment.

### 3.2. Reported Occurrence of Opacities in Caries-Free Children

The reported occurrence of opacities in caries-free children is displayed in Table 2. Among caries-free children aged 6–9 years, opacities on second primary molars were observed in less than 25% of patients (59.37%, 187/315). In total, 18% (57/315) of respondents observed no opacity in any patients, while 14.92% (47/315) of dentists noted opacities in 26–50% of the children.

Within the subgroup with opacities on second primary molars, the presence of opacities on first permanent molars were reported by 59.68% (188/315) of dentists. Opacities on first permanent molars were observed by 43.62% (82/188) of dentists in 1–25% of patients, by 30.85% (58/188) of respondents in 26–50% of patients, and by 25.53% (48/188) of dentists in 51–75% of children.

Among caries-free patients without opacities on second primary molars, the presence of opacities on first permanent molars were reported by 49.52% (156/315) of respondents. Within this subgroup, the percentage of opacities on the first permanent molars was reported by 73.72% (115/156) of dentists in 1–25% of children, by 8.97% (14/156) in 26–50% of children, and by 17.31% (27/156) of dentists in 51–75% of children.

The difference in the reported occurrence of opacities on the first permanent molars in patients with opacities on the second primary molars vs. patients without opacities on the second primary molars was statistically significant (*p* < 0.001) (Table 3).

## 4. Discussion

As far as we know, this is the first study to assess the awareness levels of dental practitioners in Northern Italy regarding MIH and HSPMs, while also exploring their perceptions of occurrence of these conditions.

While previous surveys have explored dentists’ perspectives and clinical approaches towards MIH [26,27,28], there is a notable gap in understanding dentists’ knowledge and perceptions regarding HSPMs. Existing studies [1,2,8,14,16,19,20,21,24] have predominantly focused on the prevalence and etiology of HSPMs, with comparatively little attention to clinicians’ perceptions. Assessing Italian dentists’ perceptions of HSPMs could reveal knowledge deficiency and highlight the need for further training, providing insights on existing clinical challenges.

A recent study [29] evaluated the knowledge of dental and dental hygiene students in Italy concerning developmental defects of enamel (DDE), without focusing on any specific condition. They identified gaps in students’ knowledge and management of DDE, concluding that there is a strong need to enhance education on the diagnosis and treatment of these conditions during their training. While their research provided valuable insights into the preparedness of students and highlighted the need for improved educational strategies, it did not specifically address the awareness or clinical practices related to MIH and HSPMs among practicing professionals.

Our study aims to fill this gap by exploring the perceptions and management approaches of Northern Italian dentists, who are already qualified and practicing. Investigating how Italian dentists perceive HSPMs could uncover gaps in knowledge and emphasize the necessity for additional training,

Structural anomalies, such as HSPMs and MIH, exert diverse impacts on dentition, with variable severity based on the timing of the disrupting factor during odontogenesis. Early recognition of these anomalies is crucial for effective clinical management and improving patient outcomes. Despite this importance, under-diagnosis remains pervasive [1,2,3]. Timely identification is essential for achieving treatment objectives, which include alleviating symptoms and restoring affected teeth aesthetically, morphologically, and functionally [4,5,6,7].

This questionnaire-based survey targeted dental practitioners across Northern Italy, administered through Google Forms. This study opted not to collect detailed demographic data, such as age, gender, or specialty, in order to streamline the survey. This choice restricted our capacity to contextualise the findings across various practitioner profiles and expertise levels, representing a study limitation.

Among respondents, paediatric patients constituted a limited segment of clinical practice, with most falling within the 1–25% range. This demographic composition may have influenced the study’s representativeness and the likelihood of identifying HSPM and MIH defects, potentially leading to underestimated prevalence rates.

The survey focused on caries-free children aged 6–9 years to specifically assess enamel defects. The prevalence of caries-free children in this study was lower than the WHO’s target of 80%, highlighting challenges in achieving uniform oral health outcomes across diverse populations and regions [30,31,32,33,34].

Regarding routine follow-up examinations, while a majority adhered to semi-annual recall schedules, a significant proportion conducted annual recalls or attended to patients only when urgent care was required. Diverse recall practices emphasise the need for a nuanced approach to ensure timely identification and management of oral health issues, especially in paediatric populations [35,36,37,38].

This survey highlighted that HSPM was observed among caries-free children in the range of 1 to 25%, consistent with literature findings reporting an HSPM occurrence of approximately 6.8% [39]. Limited surveys have explored general dentists’ recognition of HSPMs. A recent study from the Republic of Ireland suggested a high level of awareness and confidence in diagnosis among general dentists [40].

Interestingly, a majority of respondents noted that children with HSPMs also exhibited affected first permanent molars. The survey data highlighted that up to half of the children with HSPMs had affected first permanent molars, underscoring the intricate interplay between HSPMs and MIH. Conversely, in about 40.32% of reported cases of HSPMs, defects did not extend to involve the first permanent molars. In the absence of HSPMs, 73.72% of respondents reported encountering opacities on permanent molars in approximately 1–25% of patients.

These findings shed light on the complex relationship between HSPMs and MIH, showcasing diverse presentations of enamel defects in the paediatric population. Although several surveys have shown awareness of MIH, such as a recent study among Libyan dentists [41], awareness of the association between MIH and HSPMs remains underexplored. Recent reviews have highlighted significant variability in the prevalence and presentation patterns of these defects, emphasizing the need for standardised investigation protocols. Studies by Butera et al. reported a prevalence range of 2.9–44% for MIH and 0–21.8% for HSPMs [14]. The data on the reported occurrence from our survey align with the ranges reported in other studies [23,42] and suggest a general awareness of the association between MIH and HSPMs among Northern Italian dentist.

HSPMs have been proposed as a potential predictive factor for MIH development, with reported co-occurrence rates ranging between 11 and 20% [19,43,44]. A systematic review by Garot et al. demonstrated a higher likelihood of MIH in individuals with HSPMs [19]. Conversely, a study involving healthy children and those with medical conditions suggested that HSPMs alone may not reliably predict MIH due to potential systemic factors and medication use [24]. Moving forward, future research should further explore this relationship to enhance understanding of enamel defects and their predictive value [45,46].

A few shortcomings are noticeable in this study and deserve careful consideration. The simplicity of our questionnaire, while facilitating participation, may have restricted the depth of our analysis by omitting critical criteria to assess severity, distribution, or other clinical indicators related to enamel defects. Firstly, the lack of standardised diagnostic criteria for identifying and classifying enamel anomalies may have influenced the perceptions about MIH and HSPM occurrence. The variability in how participants assessed and categorised enamel defects could have introduced inconsistencies in our findings. Additional judgment criteria for MIH assessment, such as post-eruptive breakdown, atypical restorations, or extractions, could have provided a more comprehensive evaluation of MIH cases. By focusing exclusively on affected molars, our survey may have overlooked the presence of other enamel defects involving different teeth or presenting in different forms. Moreover, restricting the survey to patients without cavities could have biased the estimation of MIH and HSPM prevalence, potentially excluding relevant cases present in patients with cavities. Moreover, the low response rate observed in our survey may have impacted the representativeness of our sample, raising questions about the generalizability of our findings to the broader dental practitioner population.

Despite limitations, this study provides valuable insights into Northen Italian dentists’ perceptions of MIH and HSPMs. Future studies should address these limitations by incorporating demographic data and comprehensive clinical criteria to enhance understanding of dental practitioners’ perceptions and decision-making processes regarding MIH and HSPMs.

## 5. Conclusions

The findings from this survey provide valuable insights into the awareness of MIH and HSPMs among dental practitioners in Northern Italy. The data indicate that a significant percentage of dentists recognise the presence of these conditions and their potential co-occurrence in children aged 6 to 9 years.

## Figures and Tables

**Table 1:** Characteristics of respondents.

	*n* (%)
Number of respondents	315/1000 (31.50%)
Number of paediatric patients annually treated	
0–100	263 (83.49)
101–200	36 (11.43)
>200	16 (5.08)
Percentage of paediatric patients annually treated	
1–25%	259 (82.22)
26–50%	16 (5.08)
51–75%	13 (4.13)
76–100%	27 (8.57)
Percentage of children aged 6–9 among paediatric patients annually treated	
0–25%	174 (55.24)
26–50%	128 (40.63)
51–75%	8 (2.54)
76–100%	5 (1.59)
Percentage of caries-free children aged 6–9	
None	33 (10.48)
1–25%	226 (71.75)
26–50%	33 (10.48)
51–75%	18 (5.71)
76–100%	5 (1.59)
Frequency of scheduled check-up visits for children aged 6–9	
yearly	57 (18.10)
every six months	151 (47.94)
every three months	75 (23.81)
not periodically but only out of necessity	32 (10.16)

**Table 2:** Reported occurrence of opacities in caries-free children.

	*N* (%)
Percentage of opacities on second primary molars	
None	57 (18.10)
1–25%	187 (59.37)
26–50%	47 (14.92)
51–75%	19 (6.03)
76–100%	5 (1.59)
Presence of opacities on the first permanent molars, in patients with opacities on the second primary molars	
Yes	188 (59.68)
No	127 (40.32)
Percentage of opacities on the first permanent molars in patients with opacities on the second primary molars	
1–25%	82 (43.62)
26–50%	58 (30.85)
51–75%	48 (25.53)
76–100%	0 (0)
Presence of opacities on the first permanent molars, in patients without opacities on the second primary molars	
Yes	156 (49.52)
No	159 (50.48)
Percentage of opacities on the first permanent molars in patients without opacities on the second primary molars	
1–25%	115 (73.72)
26–50%	14 (8.97)
51–75%	27 (17.31)
76–100%	0 (0)

**Table 3:** Reported occurrence of opacities on the first permanent molars in patients with opacities on the second primary molars vs. patients without opacities on the second primary molars.

	Reported Occurrence of Opacities on the First Permanent Molars, in Patients with Opacities on the Second Primary Molars	Reported Occurrence of Opacities on the First Permanent Molars, in Patients without Opacities on the Second Primary Molars	
	*n* (%)	*n* (%)	*p*-Value
	188 (59.68)	156 (49.52)	
1–25%	82 (43.62)	115 (73.72)	0.00001 * (OR 0.28)
26–50%	58 (30.85)	14 (8.97)	0.00001 * (OR 4.53)
51–75%	48 (25.53)	27 (17.31)	0.06 (OR 1.64)
76–100%	0 (0)	0 (0)	

* Significant (*p*-value < 0.05).

## Data Availability

The data used to support this study are available from the corresponding author upon request.

## Supplementary Materials

The following supporting information can be downloaded at https://www.mdpi.com/article/10.3390/dj12080271/s1: Questionnaire 1: Epidemiologic survey on HSPMs (hypomineralised second primary molars) and MIH (molar incisor Hypomineralisation).
