# Supplementary material for: Awareness of Molar Incisor Hypomineralisation (MIH) and Hypomineralised Second Primary Molars (HSPMs) among Northern Italian Dentists: A Questionnaire Survey
Source: Dent J (Basel). 2024 Aug 22;12(8):271. doi: 10.3390/dj12080271 (PMC11353035; doi:10.3390/dj12080271)
Supplement: Supplementary file 1 [file dentistry-12-00271-s001.zip › dentistry-3124808-supplementary.pdf]

Supplementary Materials

Questionnaire 1 (English)

Epidemiologic survey on HSPM (Hypomineralised Second Primary Molars) and MIH (Molar Incisors Hypomineralization)

1. How many pediatric patients are in treatment in your clinical pratice?
   a. 0-100
   b. 101-200
   c. >200
2. What is the percentage of pediatric patients compared with the total number of patients?
   a. 1-25%
   b. 26-50%
   c. 51-75%
   d. 76-100%
3. What is the percentage of children aged 6-9 years among your pediatric patients?
   a. 0-25%
   b. 26-50%
   c. 51-75%
   d. 76-100%
4. What is the percentage of caries-free children among your patients between 6 and 9 years?
   a. None
   b. 1-25%
   c. 26-50%
   d. 51-75%
   e. 76-100%
5. What is the frequency of scheduled check-up visits for your patients between 6 and 9 years?
   a. Yearly
   b. Every six months
   c. Every three months
   d. Not periodically, but only out of necessity
6. Of the 6- to 9-year-old caries free patients, how many exhibit opacities on deciduous second molars (opacities: chalky, white, yellow, beige, and/or brown spots)?
   a. None
   b. 1-25%
   c. 26-50%
   d. 51-75%
   e. 76-100%
7. Among 6- to 9-year-old caries-free patients who have opacities of the deciduous second molars, do the permanent first molars, when present, also exhibit opacities (opacities: chalky, white, yellow, beige, and/or brown spots)?
   a. Yes
   b. No
8. If Yes, in what percentage?
   a. 1-25%
   b. 26-50%
   c. 51-75%
   d. 76-100%
9. Among 6- to 9-year-old caries-free patients who DO NOT HAVE opacities of the deciduous second molars, do the permanent first molars, when present, exhibit opacities (opacities: chalky, white, yellow, beige, and/or brown spots)?
   a. Yes
   b. No
10. If Yes, in what percentage?
   a. 1-25%

b. 26-50%
c. 51-75%
d. 76-100%

Questionnaire 2 (Italian)

Indagine epidemiologica su HSPM (ipomineralizzazione dei secondi molari decidui) e MIH (ipomineralizzazione di molari-incisivi)

1. Quanti pazienti pediatrici sono in trattamento nella tua pratica clinica?
   a. 0-100
   b. 101-200
   c. >200
2. Quale è la percentuale di pazienti pediatrici rispetto al numero totale di pazienti ?
   a. 1-25%
   b. 26-50%
   c. 51-75%
   d. 76-100%
3. Quale è la percentuale di bambini di età compresa tra 6 e 9 anni tra i tuoi pazienti pediatrici?
   a. 0-25%
   b. 26-50%
   c. 51-75%
   d. 76-100%
4. Quale è la pecentuale di bambini senza carie tra i tuoi pazienti di età compresa tra 6 e 9 anni?
   a. Nessuno
   b. 1-25%
   c. 26-50%
   d. 51-75%
   e. 76-100%
5. Qual è la frequenza delle visite di controllo programmate per i tuoi pazienti di età compresa tra 6 e 9 anni?
   a. Annuale
   b. Ogni 6 mesi
   c. Ogni 3 mesi
   d. Non periodicamente ma solo in caso di necessità
6. Dei pazienti di età compresa tra 6-9 anni senza carie, quanti presentano opacità sui secondi molari decidui (opacità: machcie gessose, bianche, gialle, beige e/o marroni)?
   a. Nessuno
   b. 1-25%
   c. 26-50%
   d. 51-75%
   e. 76-100%
7. Tra i pazienti di età compresa tra 6 e 9 anni senza carie e con opacità sui secondi molari decidui, i primi molari permanenti, quando presenti, presentano anche opacità (opacità: machcie gessose, bianche, gialle, beige e/o marroni)?
   a. Sì
   b. No
8. In caso di risposta affermativa, in quale percentuale?
   a. 1-25%
   b. 26-50%
   c. 51-75%
   d. 76-100%
9. Tra i pazienti di età compresa tra 6 e 9 anni senza carie e senza opacità sui secondi molari decidui, i primi molari permanenti, quando presenti,

presentano opacità (opacità: macchie gessose, bianche, gialle, beige e/o marroni)?

   a. Sì
   b. No

10. In caso di risposta affermativa, in quale percentuale?
    a. 1-25%
    b. 26-50%
    c. 51-75%
    d. 76-100%
